# Can deepfakes be used to study emotion perception? A comparison of dynamic face stimuli

**DOI:** 10.3758/s13428-024-02443-y

**Published:** 2024-06-04

**Authors:** Casey Becker, Russell Conduit, Philippe A Chouinard, Robin Laycock

**Affiliations:** 1 RMIT University, Melbourne, Australia; 2 La Trobe University, Melbourne, Australia

**Keywords:** Face perception, Generative AI, Deepfake, Emotion, Dynamic faces

## Abstract

Video recordings accurately capture facial expression movements; however, they are difficult for face perception researchers to standardise and manipulate. For this reason, dynamic morphs of photographs are often used, despite their lack of naturalistic facial motion. This study aimed to investigate how humans perceive emotions from faces using real videos and two different approaches to artificially generating dynamic expressions – dynamic morphs, and AI-synthesised deepfakes. Our participants perceived dynamic morphed expressions as less intense when compared with videos (all emotions) and deepfakes (fearful, happy, sad). Videos and deepfakes were perceived similarly. Additionally, they perceived morphed happiness and sadness, but not morphed anger or fear, as less genuine than other formats. Our findings support previous research indicating that social responses to morphed emotions are not representative of those to video recordings. The findings also suggest that deepfakes may offer a more suitable standardized stimulus type compared to morphs. Additionally, qualitative data were collected from participants and analysed using ChatGPT, a large language model. ChatGPT successfully identified themes in the data consistent with those identified by an independent human researcher. According to this analysis, our participants perceived dynamic morphs as less natural compared with videos and deepfakes. That participants perceived deepfakes and videos similarly suggests that deepfakes effectively replicate natural facial movements, making them a promising alternative for face perception research. The study contributes to the growing body of research exploring the usefulness of generative artificial intelligence for advancing the study of human perception.

## Introduction

The social messages and emotions conveyed by faces in everyday interactions are inherently dynamic, which likely explains the distinct behavioural and neural responses that dynamic faces elicit compared to static faces (Krumhuber et al., 2013; Pitcher et al., 2011; Pitcher et al., 2014). Using dynamic stimuli in human perception research is therefore essential for accurately interpreting how people perceive emotions and social interactions. However, standardising videos for this research is challenging due to factors such as variations in lighting, angle, and timing, as well as inherent variability in the spatiotemporal dynamics of a human’s expressive movements. These factors can introduce confounds that risk complicating comparisons across studies. Thus, researchers are prompted to explore alternative methods for creating consistent, standardised dynamic face stimuli.

### Controlling face dynamics

One such alternative is the dynamic morph, which artificially generates movement between two photographs, such as a neutral and peak expression. Dynamic morphs allow researchers to eliminate the impact of blinks and control factors like speed and rising duration across all portrayed actors. However, while video recordings exhibit asynchronous and non-linear motion of facial expression features (Bartlett et al., 2006; Fiorentini et al., 2012; Pantic & Patras, 2006; Zhao et al., 2016), dynamic morphs created from video recordings depict linear motion of facial landmarks, failing to capture naturalistic facial motion (Korolkova, 2018). The sequential nature of facial expressions is demonstrated by the fact that in a transition between two emotions, humans have increased awareness of the emotion that came second in real time, even when the video is played in reverse (Reinl & Bartels, 2015). Time-reversed video recordings also elicit distinct neural responses, regardless of the direction of emotion change (Reinl & Bartels, 2015). In contrast, emotion recognition for dynamic morphs is unaffected by such reversal, as they do not contain information about the flow of time (Korolkova, 2018). In addition, studies manipulating the trajectories of expression features have shown that compared to naturalistic facial motion, people perceive linear and synchronous emotion transitions as less natural (Cosker et al., 2010; Curio et al., 2006), trustworthy (Krumhuber et al., 2007), typical, intense, and genuine (Wallraven et al., 2008).

Another alternative involves using avatars or 3D models, which offer greater experimenter control over facial expressions, head position, expression duration, and blink timing (Malek et al., 2019). However, a recent meta-analysis suggests that people's responses, including emotion labelling and perception of human-likeness, tend to differ when interacting with synthetic faces compared to real ones (Miller et al., 2023). This difference might be due to reduced facial information (Crookes et al., 2015) or diminished social salience (Katsyri et al., 2020). Miller et al. (2023) noted an intriguing trend: as computer-generated faces became less realistic, participants rated interactions with them as more akin to those with real humans. The authors attributed this to several factors. First, they suggest that people may extend a kind of empathy toward less realistic, non-human agents, much as they do with pets. Second, they suggest that less realistic avatars are perceived as being incapable of forming personal judgments, which participants may find more comfortable. Lastly, they suggest that less realistic faces may circumvent the unsettling 'uncanny valley' effect, which refers to the discomfort people experience when encountering human-like entities that are almost, but not quite, lifelike (Mori et al., 2012). Faces that fall into the uncanny valley are less liked (Thepsoonthorn et al., 2021), evoke unique eye movement patterns (Cheetham et al., 2013; Thepsoonthorn et al., 2021), and elicit distinct neural responses in humans (Vaitonytė et al., 2022). Given that people do not perceive avatars and 3D models as "real" faces, the emotional and neural responses they elicit may not accurately represent the intended phenomenon being measured, highlighting the need for continued research and development of more suitable alternatives.

### Deepfakes in face perception research

The ideal solution would be a dynamic, customizable face that retains the naturalistic movement of a video recording. Deepfake technology might provide such a solution (Becker & Laycock, 2023). Deepfakes are artificially generated videos that convincingly replace a person's face or alter their appearance using advanced machine learning techniques. Unlike dynamic morphs, high-quality deepfakes can capture naturalistic facial motion (Welker et al., 2020). Recently, deepfakes have garnered attention for their potential use in face perception research (Becker & Laycock, 2023). For example, Vijay et al. (2021) used deepfake technology to manipulate the presence of eye-contact, smiling and nodding, thus experimentally isolating their impact. Deepfakes have also been employed to alter a teacher's physical attractiveness to examine student perceptions (Eberl et al., 2022). In another study, deepfakes were utilized to change the race of speakers to investigate the impact of race on credibility perception (Haut et al., 2021). This approach has previously been studied using photographs (Trawinski et al., 2021). However, dynamism may also be an important factor in the perception of race (Weisbuch et al., 2009). Indeed, results showed that while changing the race of a photograph had no difference in perceptions, deepfake race manipulation did: speakers who appeared to be white were perceived as more credible and elicited more positive sentiments. Barabanschikov and Marinova (2022) used deepfakes to create dynamic face illusions. These illusions include the Thatcher effect, where the inversion of facial features is barely noticeable when the face is upside-down but strikingly grotesque when right-side up and is used to study configural face processing. The dynamic versions produced stronger responses compared to static counterparts, providing a more robust tool for understanding the intricacies of human face perception.

### Human detection of deepfakes

What captivates and concerns us about the deepfake as a general phenomenon is that we are often unable to distinguish them from real videos. This quality also suggests their potential value as a dynamic face stimulus for psychological and cognitive neuroscience research purposes. However, using deepfake detection studies as a measure of their overall effectiveness is difficult, as many focus on the deepfakes of well-known individuals in the context of fake news (Appel & Prietzel, 2022; Dobber et al., 2021; Hwang et al., 2021; Vaccari & Chadwick, 2020). In addition, the quality of deepfakes can vary substantially. For example, one study showed that detection accuracy ranged from 24% for high-quality deepfakes to 71% for lower-quality deepfakes (Korshunov & Marcel, 2021). Other studies have shown similar detection ranges (Köbis et al., 2021; Tahir et al., 2021; Wöhler et al., 2021). Interestingly, correct identification of videos as true recordings ranges between 60 and 75%, which is similar to the rate at which high-quality deepfakes are incorrectly labelled as genuine (Köbis et al., 2021). The ability to recognise deepfakes appears to come from holistic face viewing rather than attention to fine-grained artifacts, as faces that are upside-down, misaligned, or partially obscured, reduce our ability to detect deepfakes (Groh et al., 2022). However, the strategies employed by viewers in detecting deepfakes changes depending on their quality (Tahir et al., 2021). Fortunately, factors that contribute to detection, such as blurring and asynchronous mouth movements (Thaw et al., 2021) are becoming increasingly easier to avoid as technology improves. One study showed that truncating longer deepfakes to 3 s did not impact manipulation recognition, suggesting conscious identification occurs rather early (Wöhler et al., 2021).

### The perception of deepfake emotions

Despite the high relevance of emotional expressions in human communication (Jack & Schyns, 2015), and the role of emotion in how people perceive fake news (Martel et al., 2020), few studies have examined the ability for deepfakes to accurately portray emotional expressions. Wöhler et al. (2021) did so using face swaps, which are videos in which the inner face has been replaced with a deepfake of another identity. From a longer video of an interview, a 3-s clip was extracted that portrayed one of ten emotions. A face-swap version of each video was created, and participants were informed that some clips were manipulated. Participants were worse at recognising disgust from face swaps compared to original videos, while recognition was largely similar for other emotions. Compared to original videos, people perceived face-swaps of cluelessness, disgust, and surprise as less intense, while they perceived ‘disagreement’ and ‘thinking’ as less sincere. However, participants perceived most basic emotions, such as happiness, fear, sadness, and anger, similarly for deepfakes and videos.

### Puppetry deepfakes for emotion perception research

Computational deepfake detection techniques use algorithms to detect artificially created or altered videos. These techniques often rely on discrepancies between the body/head of the underlying person, and the deepfake face that is superimposed onto the original video. These include quality differences, where the inner face appears blurred or pixelated compared to the surrounding video (Younus & Hasan, 2020), differences in skin tone or texture between the original and new face (Ajoy et al., 2021), and inconsistent blending at the contours of the face (Shao et al., 2022). Similar artifacts are reported by humans who correctly identify deepfakes (Wöhler et al., 2021). Movement discrepancies can also serve as indicators of deepfakes. For example, subtle spatiotemporal discontinuities may occur, where the position of the inner face shifts slightly throughout the video (Yu et al., 2023). The movement of the ears, which belong to the underlying identity, can be used to detect movement discontinuities in face-swap deepfakes (Agarwal & Farid, 2021). This may impact the perception of emotions from face-swaps, as subtle head movements are involved in emotional expressions (Atkinson et al., 2004; Busso et al., 2007; Livingstone & Palmer, 2016; Tracy & Matsumoto, 2008; Tracy & Robins, 2004).

**Fig. 1:**
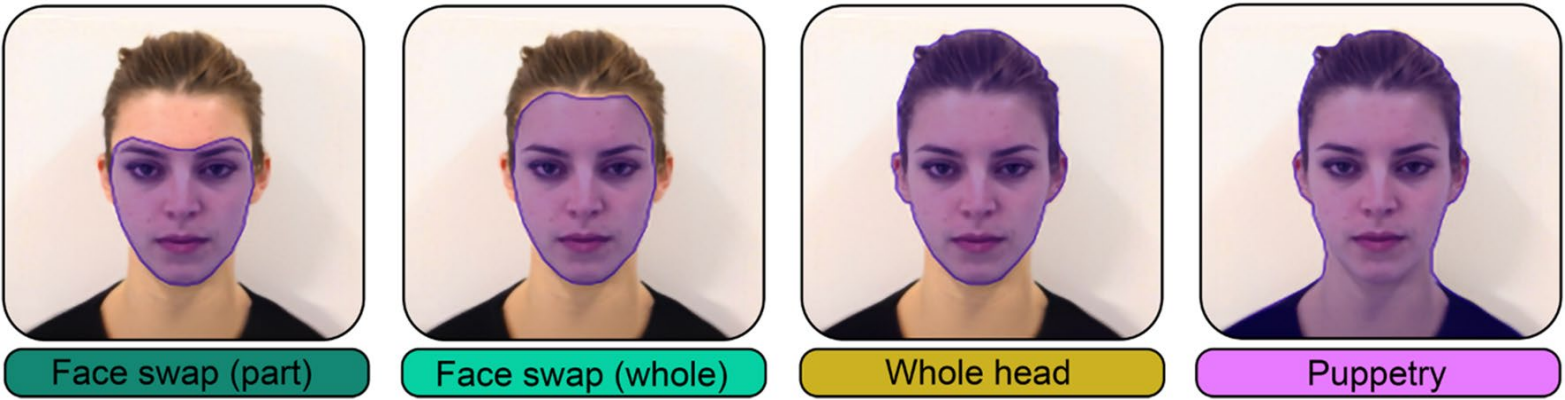
Mask placement on deepfake (DF) types. *Note. Shaded areas* indicate the area that will be replaced with another identity. Face-swaps are the most common form of deepfakes, and require advanced mask blending techniques. Whole-head and puppetry deepfakes require all training images to portray the same hairstyle. Puppetry deepfakes replace all visible features with another identity and are the only deepfake type that does not require similar skin tones

It is also possible to construct fully synthesised deepfakes, which replace all visible features of a person. These “puppetry” deepfakes transfer all identity features (including hair, neck, and shoulders), onto the temporal characteristics of the destination video (see Fig. 1). These more complete deepfakes avoid issues relating to colour transfer, imperfect facial alignment, and mask edge artifacts. The impact of using fully synthesized deepfakes, rather than videos with only the inner face synthesised (as in Wöhler et al., 2021) on how we perceive emotion perception remains unclear. While Wöhler et al. (2021) provide a useful database, we aimed to determine the ability for deepfakes to produce the types of expressions frequently employed in face perception research. Thus, we created tightly controlled, face-forward, close-up deepfakes of emotional expressions using a well-validated dataset of neutral-to-peak expressions.

### The perception of deepfakes in naïve viewers

While explicit deepfake detection is important for improving techniques and developing countermeasures against malicious use, perhaps more critical to the field of face perception is how deepfakes are perceived by naïve viewers (that is, participants who have not been informed that the dynamic face is a deepfake). Wöhler et al. (2021) examined eye gaze behaviour in naïve participants and found that participants looked more towards the mouth for deepfakes, but only those which had visible artifacts. Social judgements of deepfake emotions in naïve viewers remains unclear.

## The current study

We aimed to evaluate how well dynamic morphs and deepfakes could convey the emotions captured in the original video recordings from which they were created. Based on findings from research comparing naturalistic and unnatural facial motion, we expected that dynamic morphs would be perceived as less intense and genuine compared to videos (Wallraven et al., 2008). Given Wöhler et al. (2021) showed similar intensity (i.e., strength) and sincerity (i.e., genuineness) for most basic emotions depicted in face-swap deepfakes, we expected similar results for whole-head puppetry deepfakes. Additionally, we gathered qualitative data on whether participants found anything unusual about three types of stimuli through post-experiment questionnaires.

## Method

### Participants

A power analysis was conducted aiming for 95% power, using an effect size of *f* = 0.68 from Wöhler et al. (2021). We aimed to double the required sample size to allow for gender-based analysis, given that different genders may perceive faces differently (Forni-Santos & Osório, 2015), and sex/gender disaggregated data is encouraged in psychological science (Lefler et al., 2023). In addition, we wished to better account for the likely small difference between face-swaps and puppetry deepfakes, as the latter are expected to be more similar to original videos. Participants were recruited via online advertisements posted in 2022 via multiple platforms, including Facebook, Twitter, Instagram, LinkedIn, and Reddit. The study information did not disclose the use of manipulated media (i.e., deepfakes, morphs), as we aimed to measure the perception of emotion in naïve participants. We targeted a variety of groups such as volunteer groups, community groups, and student groups across multiple English-speaking towns and cities.

Participants interested in taking part in this study completed an online screening questionnaire, and those aged between 18 and 40 (as vision declines with age; Mateus et al., 2013) and who reported normal vision, were invited to complete the online experimental study, and provided informed consent via checkbox. Two participants were excluded as monitor sizes reported by the experiment platform indicated the use of web-mode on a mobile device. One participant was excluded due to a loading delay of more than 10 s for more than 20% of trials. No loading delays were reported for any trials for other participants. Ninety-three participants were included in the analysis (49 female, 41 male, three non-binary/gender diverse). Included participants were located in Australia (70), United States (8), New Zealand (2), United Kingdom (1), Sri Lanka (1), Vietnam (1), Georgia (1), and Malaysia (1) (unknown: 8).

Thirteen participants indicated that they had a diagnosis of a psychiatric or neurological condition. All thirteen of these participants chose to list one or more condition (anxiety related: 5, depression related: 4, bipolar disorders: 3, attention-deficit hyperactivity disorder: 4, obsessive–compulsive disorder: 2, personality disorder: 2, post-traumatic stress disorder: 2, neuropathic disorders: 1, epileptic disorders: 1). Due to a technical error, the exact ages of the participants were not recorded. The study was approved by the local ethics committee of RMIT University.

## Materials

### Video recorded stimuli

Facial expressions of happiness, anger, fear, and sadness were sourced from the Amsterdam Dynamic Facial Expression Set (ADFES; van der Schalk et al., 2011). While the time it takes to express spontaneous emotions typically varies, rising time for posed expressions can be similar across emotions (Mavadati et al., 2016). We observed that in the ADFES database, many of the actors took over 800 ms to transition from neutral to peak for all studied emotions (anger, fear, happiness, and sadness). This allowed us to standardise all emotion transitions to 800 ms. By cutting the videos before the expression reached peak emotion intensity, we ensured that the observed “peak” in the edited stimulus occurred in the final frame of the transition. Videos were chosen if the expression at 800 ms was still rising – i.e., videos of expressions that reached the peak too quickly were excluded. Some sad expressions that took much longer to unfold were excluded, as the expression at 800 ms was ambiguous. If one emotion was excluded, the actor was excluded, thus the actors are the same for all four emotions. The neutral frame before expression onset was presented for 200 ms (five frames at 25 fps), and the final frame of the video was held for 200 ms (five frames at 25 fps), resulting in a total stimulus time of 1200 ms (30 frames at 25 fps). These frame-holds created the illusion of a complete expression without a noticeable pause. The resulting expressions were depicted by ten actors: five female (three Mediterranean [Turkish/Moroccan], two northern European) and five male actors (one mixed-race, four northern European), thus resulting in 40 video stimuli. These videos were used to create dynamic morphs and deepfakes, thus all stimuli portrayed the same actors and emotions. Each dynamic stimulus had a framerate of 25 fps. Permission to modify and adapt the videos from van der Shalk et al (2011) was obtained through personal communication.

### Morphed expressions

**Fig. 2:**
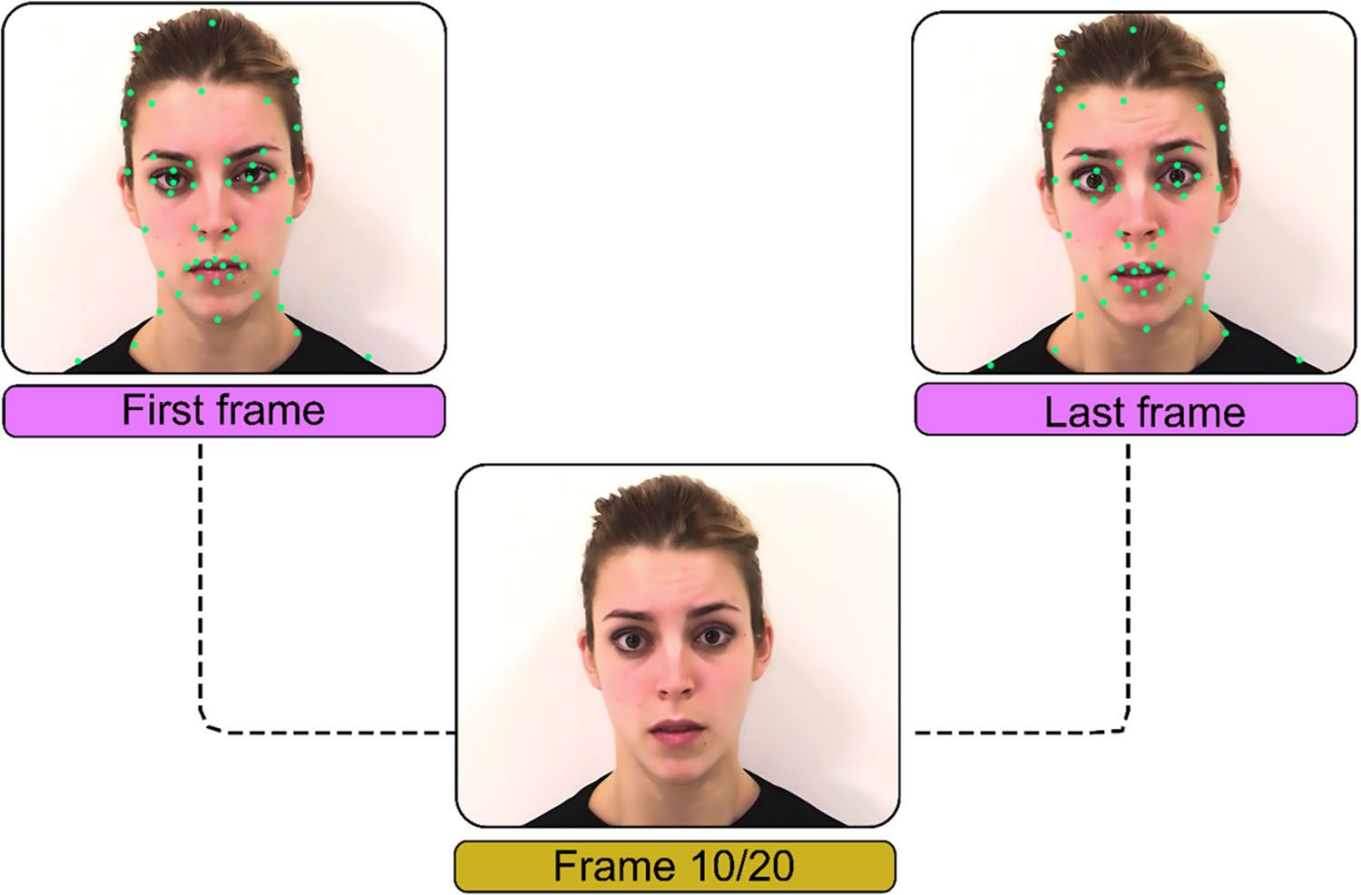
Example dynamic morph landmark placement on one actor. *Note.* Correctly placed landmarks at corresponding points on the neutral (*left*) and peak (*right*) results in a clear transition without overlap. The figure shows the resulting video frame (*lower*) that would be seen at the midpoint of the morphed video sequence

Dynamic morphs were created by using the neutral and peak expression still frames from each video stimulus (after editing to remove noise), and linearly morphed using FantaMorph© (Abrosoft, 2011; Version 4.1), which simulated movement between the two images. To create the dynamic morph, markers are placed at various points on the neutral expression, and their corresponding locations in the peak expression. Approximately 50 markers were placed at various points of the face, especially at the edges of facial features, and points with the most movement (Kaufman & Johnston, 2014). These include the pupils, eye corners, eye lids, hairline, lip edges, and facial moles (see Fig. 2). Before any markers are placed, the morph preview shows the neutral starting image with the expressive image overlaid, with increasing opacity as the video progresses. To ensure the highest-quality dynamic morph, care was taken to adjust markers until the images in each intermediate frame were aligned, showing no overlap. Morphed stimuli lasted 1200 ms, and consisted of a 200-ms static neutral, an 800-ms dynamic sequence, and 200-ms static peak.

### Creation of deepfake stimuli

**Fig. 3:**
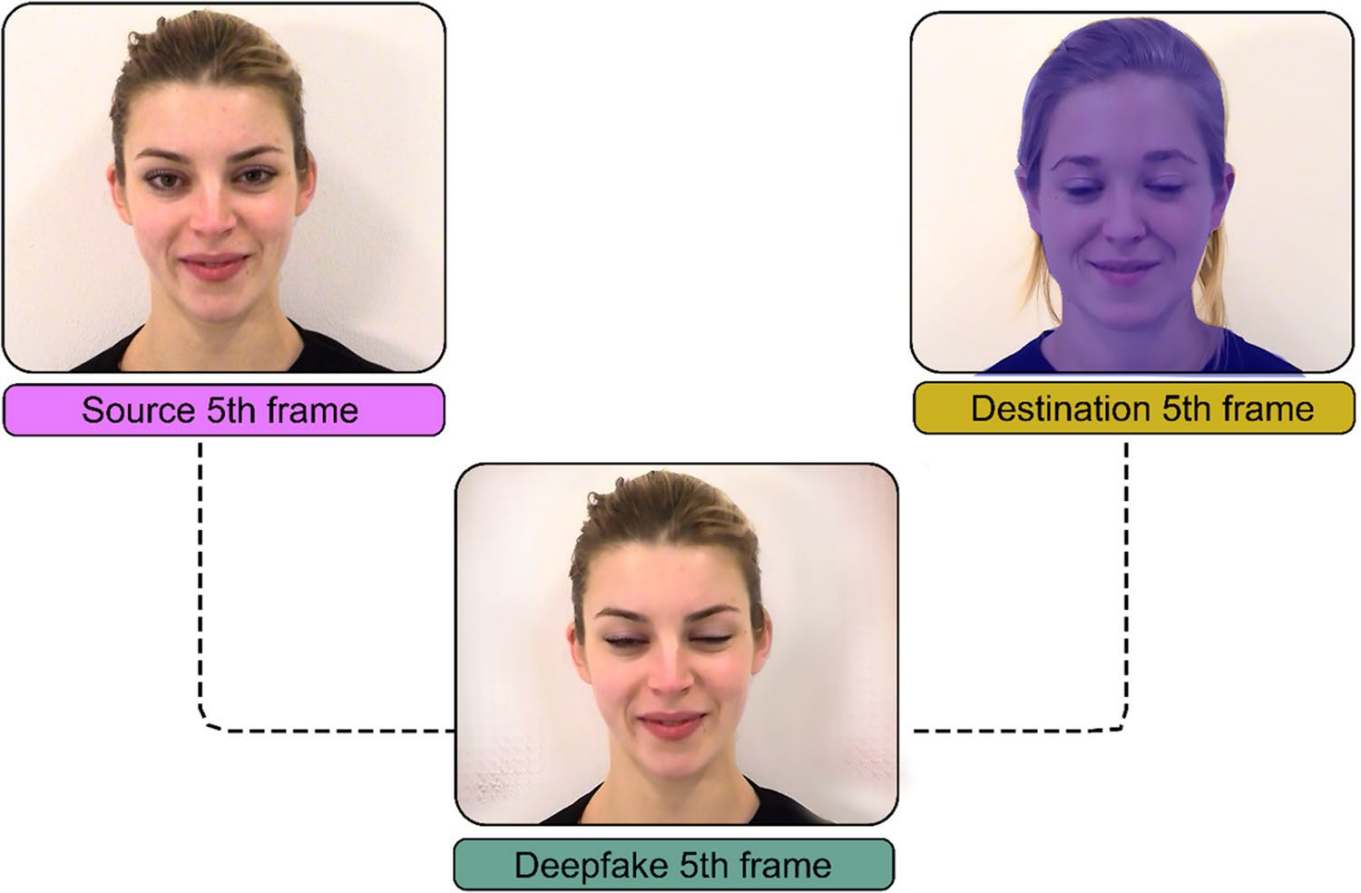
Example source and destination that contribute to a deepfake. *Note.* The final deepfake blends the identity of one video (the source) with the temporal dynamics of another video (the destination). In puppetry deepfakes, the identity in the destination remains unseen in the final deepfake. If the destination blinks at the 5th frame of an expression transition, the source identity will also blink in the 5th frame of the final deepfake

Further details on the parameters used to create deepfakes are accessed in the available online repository. Puppetry deepfakes were created with the software DeepFaceLab. For each deepfake, an autoencoder was trained on the frames extracted from two different videos of a given emotion. One video (and its extracted frames) is known as the “source” and depicts the identity that will end up in the final deepfake. The other is known as the “destination” and contains the temporal features and dynamic movements that will end up in the final deepfake. In the case of puppetry deepfakes, the identity of the destination face remains completely unseen (see Fig. 3). Source identities matched the actors in video and dynamic morph stimuli. Destination dynamics were sourced from unseen actors of the same gender as the source.

We used a whole-head DF-UD pretrained model, which is suitable for puppetry deepfakes. Model settings were chosen after exploring The DFL 2.0 Model Settings and Performance Sharing Thread and similar resources. Given that many resources are tailored toward face-swap deepfakes, alterations in popular sequences were tested until training times and output quality matched those typically reported. A brief description for each parameter used can be found in Table 1.

**Table 1:** Brief descriptions of the parameters used to create deepfake stimuli. More detailed information on parameters can be found in the online repository

Parameter	Description
Batch size	The number of faces compared per iteration; affects quality of output and speed of training.
Random warp	Randomly deforms facial features of the source image to create a distorted image; prevents overfitting.
Learning rate dropout	Randomly sets some learning rates to zero during each iteration; prevents overfitting.
Eyes/mouth priority	Captures nuances in eyes and mouth
Adabelief	Optimiser, improves accuracy and quality of output
GAN	Improves details and sharpness of faces
Gradient clipping	Prevents model collapse / corruption
True face	Preserves details of the source identity in the generated deepfake
Random flip	Randomly flips the input image; improves generalisation.

All iterations were run with the following settings: resolution: 320 pixels, autoencoder dimensions (ae_dims): 256, encoder dimensions (e_dims): 256, decoder dimensions (d_dims): 80, decoder mask dimensions (d_mask_dims): 32, batch size: 8. These dimensions were used as they generated the highest-quality output on the available hardware (Gigabyte GeForce RTX 3080 Aorus Extreme LHR 10GB).

The following settings were used throughout: masked training (true), uniform yaw (false), AdaBelief (true). For the first 50,000 iterations (or until loss values were no longer falling), the following settings were used:

*True:* random warp, random SRC (source) flip, random DST (destination) flip.

*False*: uniform yaw, LRD (learning-rate dropout) GAN (general adversarial network), true face, eye/mouth priority.

At this stage, output was checked for similarity to the source expression. Where there were expression artifacts, problematic source frames were deleted (e.g., source photos showing teeth can confuse a model trying to reproduce a closed-mouth smile), and the model was restarted. Settings were then modified according to the following sequence, with each step running for approximately 50,000 iterations, or until loss values were no longer falling: (1) eyes/mouth priority: off, (2) learning rate dropout: on, (3) learning rate dropout: off, random warp: off, (4) learning rate dropout: on, (5) GAN: on (recommended settings), (6) Trueface 0.01: on. In the case of model collapse, models were restarted at the first 50,000 iteration point. Given that the backgrounds were identical, output was generated without a mask, which retained the hair and shoulders of the source (i.e., the new identity).

### Ensuring consistency across stimulus types

Original videos contained film grain and colour noise that were absent from the deepfakes, and these features are difficult to replicate post-production. To focus on the perception of the human faces and their expressions, rather than potential non-biological cues, we used Adobe After Effects to filter noise from the video recordings. This ensured that frames looked similar between videos and deepfakes, allowing for a comparison on the realism of the facial dynamics. Such adjustments are akin to the colour and contrast corrections often used when matching static stimuli from different sources. In addition, discrepancies between the ADFES face files and the exported frame formats used by Fantamorph resulted in colour space inconsistencies. To ensure consistency across all stimuli, we matched colour and appearance across stimuli using Lumetri Scopes in Adobe After Effects, which visualises differences in various low-level image properties. All stimuli are freely available in the online repository provided.

## Experimental procedure and tasks

Participants completed the experimental task online from a laptop or desktop computer. The experiment was created using the online behavioural experiment builder Gorilla (Anwyl-Irvine et al., 2020), and ran for approximately 20 min, interspersed with short rest periods to avoid fatigue. Every participant completed 120 trials, including 40 trials for each display type (video recording, dynamic morph, deepfake), with ten trials for each emotion (happy, angry, fearful, sad).

**Fig. 4:**
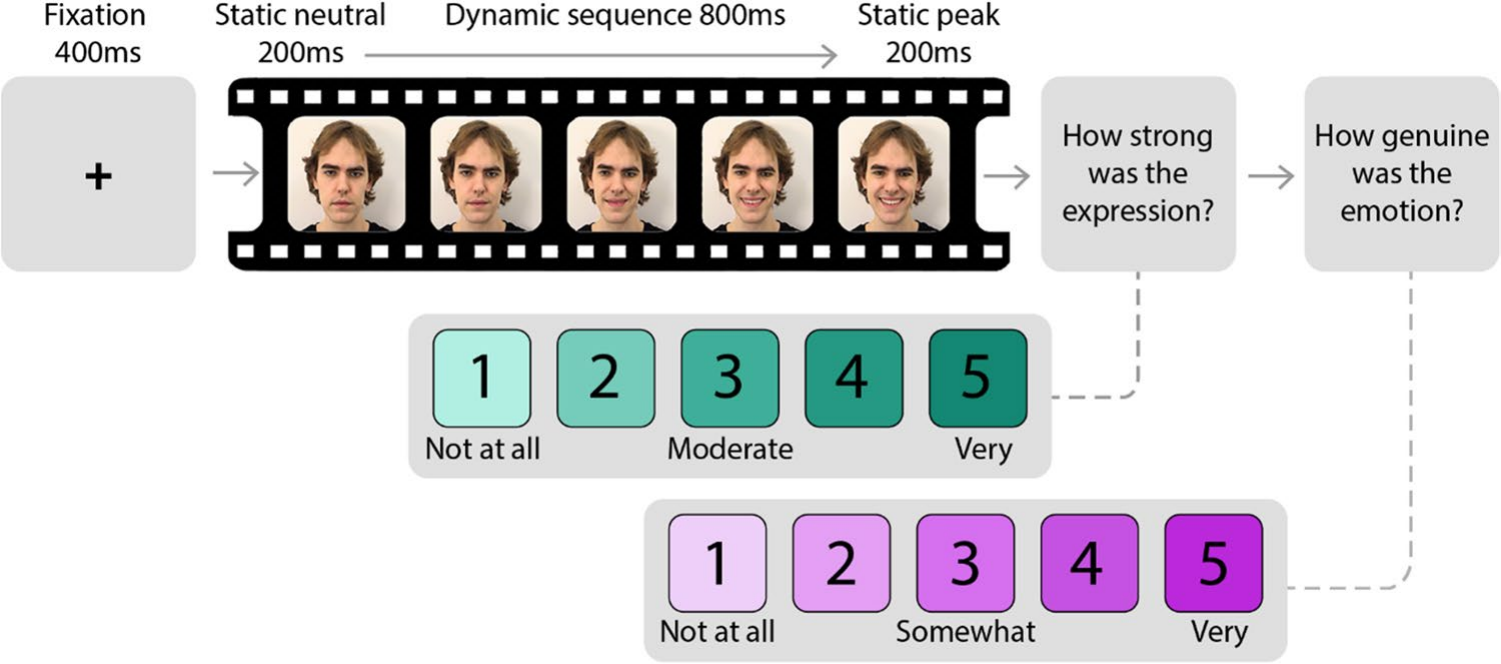
Trial sequence: 1200-ms stimulus followed by strength and genuineness questions. *Note.* The stimulus sequence included a 200-ms neutral freeze-frame, 800-ms transition, and then a 200-ms peak freeze-frame. Participants then selected a response for questions related to expression strength and then emotion genuineness

The timeline of an experimental trial is depicted in Fig. 4. Trials began with a fixation cross on a grey background, lasting for 400 ms, followed by the face expression stimulus, which lasted 1200 ms, followed by a blank screen for 500 ms. Subsequently, participants were asked “How strong is the expression” and indicated their answer using a five-point Likert scale ranging from very weak (1) to very strong (5), with a midpoint label of moderate (3) (Livingstone & Russo, 2018). Participants were then asked, “How genuine is the emotion”, and provided an answer using a five-point Likert scale with three labels: (1) Not Genuine (3) Somewhat Genuine, (5) Very Genuine (Livingstone & Russo, 2018). Note that genuineness was explicitly framed in terms of emotion, rather than as an indication of real or manipulated content, as participants were unaware the stimuli contained deepfakes or dynamic morphs. A practice block was conducted in which participants responded to three stimuli. During practice, participants were instructed that the ratings of strength and genuineness were independent from each other, and to not overthink their ratings. Each display type was presented in separate, but otherwise identical blocks, the order of which were counterbalanced across participants and interspersed with short breaks.

## Statistical analysis

For each rating type (genuineness, strength) a two-way repeated-measures ANOVA with the following factors: “display type” (video, morph, deepfake) and “emotion” (anger, fear, sadness, happiness) was performed on the mean ratings for each condition. Results were considered as significant at the level of *p* < .05. Significant main effects were further analysed with post hoc tests, and interaction effects were interpreted using simple main effects. In both cases, Bonferroni corrections were used to correct for family-wise error, and reporting of corrected *p* values are denoted with “p_c_”. Estimates of effect size are reported as partial eta squared (η^2^_*p*_), and their magnitude was interpreted using the convention: 0.0099 (small effect), 0.0588 (medium effect), and 0.1379 (large effect; Cohen, 2013). Frequentist statistical analyses were performed using the statistical software package SPSS (version 28.0).

In addition to the null hypothesis significance testing (NHST) described above, a directed Bayesian analysis was also included. Specifically, we calculated Bayes factors to test for the hypothesised null findings between video recorded and deepfake stimuli that are not possible with NHST (Rouder et al., 2009). These Bayes factors (BF01) determined the likelihood of the null hypothesis relative to the alternative. BF01 > 1 indicated evidence for the null hypothesis, with values of 1–3 providing anecdotal evidence, 3–10 offering moderate evidence, 10–30 providing strong evidence, 30–100 indicating very strong evidence, and >100 considered to present decisive evidence (Jarosz & Wiley, 2014). Bayesian statistical analyses were performed using JASP (JASP Team, 2023).

## Qualitative data analysis using large language models

At the end of the experiment, participants were asked whether they noticed anything unusual about any of the three tasks they had just completed. Participants were presented with one free text field for each task, labelled “first task”, “second task”, and “third task” (note: these were re-coded as the order was counterbalanced across participants). Eighty-one participants (87.10%) entered text into at least one field. Full data are available in the online repository provided.

Considering there are limited data on the perception of deepfakes and dynamic morphs, we aimed to conduct an inductive analysis of the qualitative data. Although combining qualitative and quantitative research methods can be beneficial, the former is typically conducted without a hypothesis (Morse & Mitcham, 2016). Inductive reasoning is data-driven, with themes and patterns emerging from the data itself rather than being dictated by pre-existing expectations or theories (Thomas, 2006). Hypotheses in qualitative analysis can result in confirmation bias and illusory correlations, where researchers are influenced by their preconceived notions of the data (Onwuegbuzie & Leech, 2007).

Given the qualitative data collected is embedded within a hypothesis-driven, quantitative design, we aimed to have our qualitative data analysed by a third party. We required that our third party be (1) blind to the study’s hypotheses and aims, (2) extensively trained in qualitative analysis of qualitative data, and (3) not have any pre-existing opinions on deepfakes or artificially manipulated media.

We used OpenAI’s fourth-generation generative pre-trained transformer GPT-4-1106, due to its unprecedented size GPT 3.5 turbo for our analysis. Similar LLMs have previously been used to analyse qualitative data (Baldwin, 2008; Crowston et al., 2012; Crowston et al., 2010; Leeson et al., 2019; McHugh et al., 2020; Nguyen et al., 2020; Ranard et al., 2016). These models can identify and categorise recurring themes and patterns in natural language text without prior knowledge of the study aims or hypotheses (Bail, 2016; Scaccia & Scott, 2021; Tounsi & Temimi, 2023), with results comparable to human analysis (Crowston et al., 2012; Fang et al., 2022; Leeson et al., 2019; Lennon et al., 2021). As the largest model to date, with a trillion parameters (AX Semantics, 2023), GPT-4 is especially well-suited for qualitative data analysis (Sen et al., 2023).

We used the API version of the model, which allows users to specify the level of randomness in the output, leading to increased accuracy and reliability (Chew et al., 2023). All qualitative data and full transcript, including follow-up questions to confirm the methods used, are available in the online repository. The GPT conversation is also available, including a follow-up question about the process used: https://platform.openai.com/playground/p/ewC3bFg15V215NMlD22hFyGS?model=gpt-4-1106-preview&mode=chat. A fresh instance of the model was given the following low-perplexity (Gonen et al., 2022) system prompt, which includes instructions to perform a thematic analysis on the qualitative data:

*You are an expert in qualitative data analysis using inductive reasoning. You will be sent comments made by experiment participants after an experimental task. Participants are responding to the question: "did you find anything unusual about this task?". Each new line corresponds to one participant's comments. You should conduct an inductive, thematic analysis – which is a qualitative method used to identify, analyse, and report patterns or themes within the data. For each task, you should carefully read the comments, generate initial codes, search for themes, and review and refine these themes. You should provide a paragraph for each task that summarises the themes that you have identified within that task, using quotes from the data as examples. Then, provide a concise summary on the key differences between each task.*

The qualitative data were then sent to the model for analysis. Paragraphs were titled Task V, M, and D, for video, morph, and deepfake, respectively. Full paragraphs are available in the online repository.

## Results

We examined potential confounding factors of gender, handedness, and clinical diagnosis in our analysis. No differences between groups were observed with gender or clinical diagnosis as a between-subjects factor (*p’*s > .05). Due to the limited number of left-handed participants, we conducted a separate analysis for this factor where those participants were excluded. The outcomes of these analyses were consistent with our main findings, indicating that handedness did not confound our results. In the following sections, we present the results from our primary analysis with all participants included, and which are substantiated by these supplementary investigations.

The assumption of sphericity was violated for both ANOVAs. Therefore, Greenhouse–Geisser correction was applied to the degrees of freedom reported.

## Strength

**Fig. 5:**
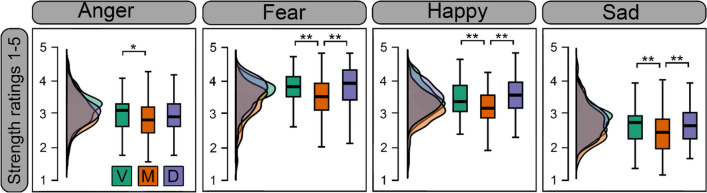
Strength ratings of expressions for each display type. *Note.* Distribution and boxplots of mean strength ratings (1–5 Likert scale) per emotion for videos (V; *green*), morphs (M; *orange*), and deepfakes (D; *purple*); *p_*c*_ < .05, **p_*c*_ < .01. Higher scores indicate higher perceived strength of the expression

Strength ratings for each condition can be seen in Fig. 5. ANOVA revealed a significant main effect of display type (F(1.60, 147.36) = 18.403, *p* < .001, ηp^2^ = .167). Participants evaluated morphs as having weaker emotion strength compared to both videos and deepfakes (*p*_*c*_*’s* <.001), while strength ratings between videos and deepfakes were not significantly different (*p* > .999). There was a significant effect of emotion (F(2.40, 221.57) = 164.174 *p* < .001, ηp^2^ = .641); fearful emotions were perceived as having the highest emotion strength, followed by happy, then angry, then sad expressions (*p*_*c*_’s < .011). Finally, there was also a significant interaction between display type and emotion (F(5.16, 474.31) = 6.28, *p* < .001, ηp^2^ =.066). For all expressions, videos and deepfakes were not significantly different (*p*_*c*_’s > .229). Deepfakes and videos were perceived as having stronger emotion than the respective expressive morph for fearful (*p*_*c*_*’s* <.001), happy (*p*_*c*_*’s* <.001), and sad expressions (*p*_*c*_*’s* <.029). The same pattern was observed for angry expressions, however after Bonferroni correction, only videos were perceived as significantly stronger than morphs (*p*_*c*_ = .019).

We expected deepfakes and videos would be similar, hence BF_01_ was used for the Bayesian paired-samples *t* test between deepfakes and videos, which tests the strength of the null hypothesis. This indicated strong evidence that video recorded and deepfake emotions were perceived as similarly intense (BF_01_ = 8.221).

## Emotion genuineness

**Fig. 6:**
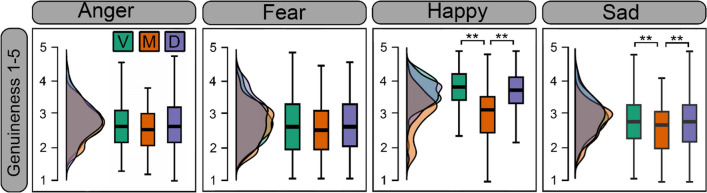
Genuineness ratings of emotions for videos (V), morphs (M), and deepfakes (D). *Note.* Distribution and boxplots of mean ratings (1–5 Likert scale) per emotion for Videos (V; *green*), morphs (M; *orange*), and Deepfakes (D; *purple*); **p < .01. Higher scores indicate higher perceived emotion genuineness

Emotion genuineness ratings for each condition can be seen in Fig. 6. ANOVA revealed a significant main effect of display type (F(1.35, 124.01) = 14.75, *p* < .001, ηp^2^ = .138). Participants evaluated dynamic morphed emotions as less genuine compared to videos (*p*_*c*_ < .001) and deepfakes (*p*_*c*_ < .001), while videos and deepfakes did not differ (*p*_*c*_ >.999). There was a main effect of emotion (F(2.4,220.74) = 99.89, *p* < .001, ηp^2^ = .521). Happiness was seen as most the genuine emotion (*p*_*c*_’s <.001), while there were no significant differences between other emotions (*p*_*c*_’s > .201). There was a significant interaction between display type and emotion (F(4.43,407.44) = 24.36, *p* < .001, ηp^2^ = .209). Morphed happiness and sadness were rated as less genuine compared to videos (*p*_*c*_’s < .019), and deepfakes of the same emotions (*p*_*c*_’s < .022). Videos and deepfakes were not significantly different for any emotion (*p*’s > .112). The perception of genuineness for anger and fear did not differ statistically between each display type (*p*_*c*_’s > .240).

We expected deepfakes and videos would be similar, hence BF_01_ was used for the Bayesian paired-samples *t* test between deepfakes and videos, indicating moderate evidence that videos and deepfake emotions were perceived as similarly genuine (BF_01_ = 7.62).

## Qualitative analysis

The following presents the ChatGPT output from the prompt:

*Task M:*

*The predominant theme that emerges from Task M is the perception of artificiality in the facial expressions presented. Participants frequently described the faces as "fake," "computer-generated," "digitally edited," or "AI imaging." Many noted that the transitions between expressions were unnatural, using terms like "morphed," "jagged," "slowed," and "unnatural." Specific aspects of the faces, such as the mouth and facial proportions, were often mentioned as manipulated or not moving naturally. Despite a few participants not noticing anything unusual, the consensus indicates a strong sense of digital interference, with comments like "faces looked warped," "expressions were more exaggerated," and "seemed like a morph between photos." The overall impression is that the task involved some form of digital manipulation, leading to expressions that were perceived as insincere or less genuine.*

*Task V:*

*In contrast to Task M, Task V elicited a mix of perceptions regarding the authenticity of the facial expressions. While some participants still sensed artificiality with comments about "fake/ai generated" clips or "computer changed" expressions, others found the task to feature "the most genuine transition" or "real videos." The theme of authenticity is more pronounced here, with several participants noting that the expressions felt natural or that the videos seemed to be of "normal length." However, there were still mentions of repetition and forced expressions, suggesting a blend of genuine and manipulated content. The speed of the videos was also a point of discussion, with some participants observing that the expressions were "sped up" or "slowed down."*

*Task D:*

*For Task D, the central theme revolves around the visual presentation of the videos, with many participants noting a "filter" or alterations in "contrast" and "lighting." The expressions themselves were often described as "stronger" or "more obvious," with a few comments on the authenticity of specific emotions like shock or anger. The notion of video manipulation is present but less focused on the expressions themselves and more on the overall aesthetic quality, with terms like "glowy," "high contrast," and "colour saturated" being used. Some participants felt the expressions were "faster" or "slower" than in previous tasks, and there were mentions of "enhancement" and "repetitions" of expressions.*

*Summary of Key Differences:*

*The key differences between the tasks lie in the perceived authenticity and type of manipulation. Task M is characterized by a strong sense of artificiality, with participants frequently noting digital manipulation of facial expressions. Task V presents a more mixed perception, with some participants sensing genuine expressions while others still detect artificiality. Task V seems to strike a balance between real and manipulated content. Task D is distinguished by comments on visual filters and alterations to the video quality, rather than the expressions themselves. The expressions in Task D are noted as stronger or more obvious, with some focus on the authenticity of specific emotions. Overall, Task M is associated with digital distortion of expressions, Task V with a blend of real and manipulated content, and Task D with visual enhancements affecting the presentation of the videos.*

**Fig. 7:**
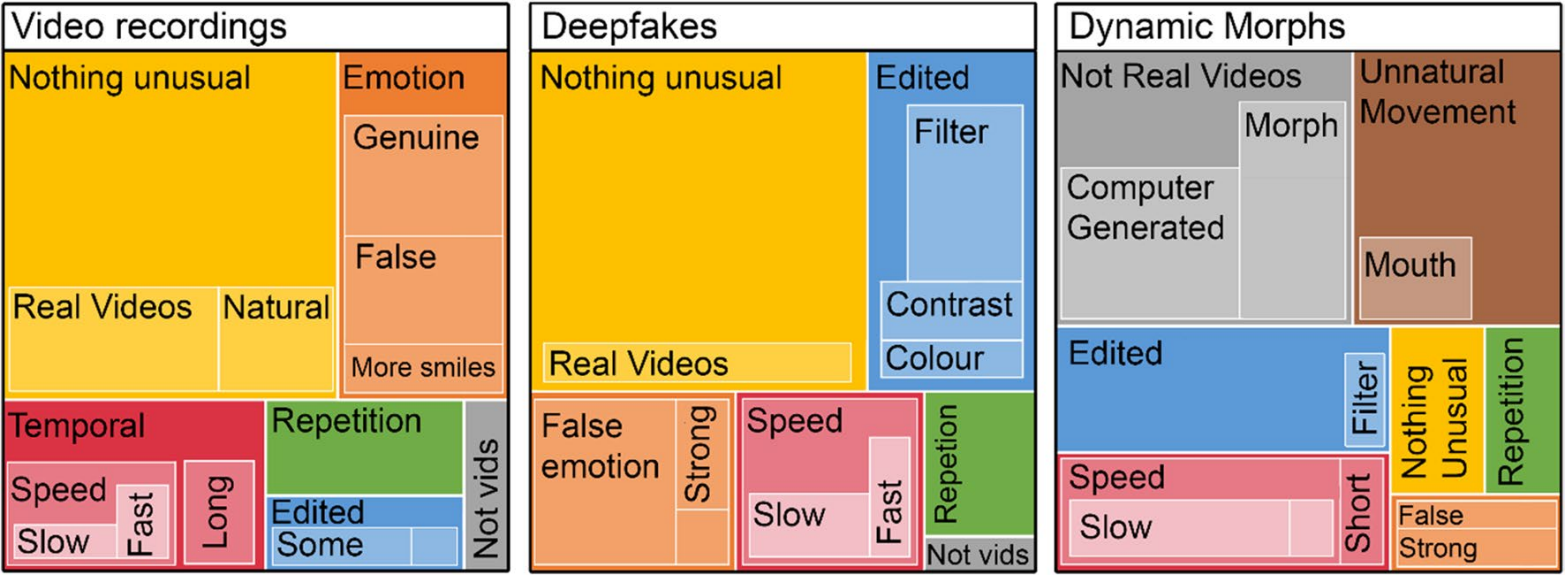
Hierarchy chart of codes for videos, deepfakes, and dynamic morphs. *Note.* Themes for each display type were coded using NVIVO qualitative data analysis software. *Larger boxes* indicate larger amounts

As the use of AI in qualitative analysis is relatively new, we conducted separate researcher analysis to confirm our findings (Guetterman et al., 2018; Leeson et al., 2019). The researcher was blind to the results generated by the AI. The researcher remained reflexive throughout the process, striving to minimize any influence from their knowledge of the study hypotheses and being open to identifying new themes that may emerge. The intention was to ensure the robustness and credibility of the GPT-4 findings by using a form of triangulation. The themes identified from this process were found to be similar to those generated by GPT-4. i.e., the researcher also found that morphs were described as computer-generated or manipulated, and that tasks V and D were similar. These themes were coded and visualised using NVIVO (Version 14) and can be seen in Fig. 7. NVIVO data and researcher summary are provided in the online repository. The congruence between analyses provides additional confidence in the study's qualitative findings, reinforcing the trustworthiness of the results. Although the potential for bias in the researcher analysis cannot be eliminated, the effort to minimize its impact, combined with the similarities in the findings from both GPT-4 and the researcher, supports the overall themes identified in this study.

instances of each theme. Subthemes are represented as a lighter coloured box within a theme.

## Post hoc analyses

Although it did not emerge as a theme, qualitative analysis revealed that some participants indicated the possibility of habituation in the video task. Thus, we analysed the average intensity for the first and second half of trials in each task. We found that deepfakes were perceived as less intense over time, but the same was not true for videos or dynamic morphs. However, simple main effects revealed the same relationship between stimulus types for the first half as for the second half of the experiment.

While morphs were made from the first and last frame of videos, the first and last frame of the corresponding deepfake are similar, but not identical. We therefore conducted a follow-up study to compare neutral and peak frames from videos (which is also the first and last frame from dynamic morphs) and deepfakes, presented for 1200 ms each. Twenty-six participants rated the first (i.e., neutral) and the last (i.e., peak) frame in separate blocks of trials using the strength Likert scale described in the study. To avoid floor effects on the neutral condition, we ran this second, so that we could adjust participant expectations. Thus, for the neutral frame condition, participants were informed that the actors were suppressing an emotion, but that some actors were better than others. They were told that this time, they should use the highest rating (5) for expressions that show a lot of emotion, given they are supposed to be neutral.

For the peak frame, deepfakes and videos/morphs had average ratings of 3.6 (*SD* = 0.49) and 3.5 (*SD* = 0.47), respectively. Bayesian analysis (BF01) revealed moderate evidence for a small difference in strength ratings. In the case of neutral frames, deepfakes averaged 2.64 (*SD* = 0.57), slightly higher than 2.49 (*SD* = 0.53) for videos, with strong evidence supporting this difference as per BF01. This effect may be due to the deepfake training data, which contained frames from the full 6-s ADFES transitions. Given that the expression begins to unfold after approximately 1 s, the training data contained more expressive frames compared to completely neutral frames. This may cause the algorithm to incorporate faint traces of the expression into the frames that are intended to be purely neutral. The final frame of our truncated deepfakes may also contain traces of the upcoming expression, which may become slightly more intense for some actors. It is important to note that these frames were showed for 1200 ms in the follow-up experiment, but only appeared for 200 ms in the original dynamic task. Interestingly, even though there were slight differences in the static frames between videos and deepfakes, participants viewed the equivalent dynamic deepfakes/videos as having similar emotion strength. We therefore argue that differences in dynamic movement of deepfakes, rather than differences in their first or last frame, explains why they outperform dynamic morphs.

## Discussion

This study aimed to compare people’s perceptions for real video recorded expressions with dynamic morphs and deepfakes, both of which were created from the same original videos. Participants found videos and deepfakes similarly genuine in expressing emotion, both in intensity and genuineness, while dynamic morphs, despite sharing the same initial and final frames as videos, were seen as less intense and genuine. For emotion intensity, this pattern was driven by happy, fearful, and sad expressions. For angry expressions, only videos were stronger than dynamic morphs. For emotion genuineness, the main effect was driven by happy deepfakes and videos, as well as sad videos, as participants perceived these as more genuine compared to dynamic morphs of the same emotions. Anger and fear were seen as similarly genuine across all display types. When participants were asked if they noticed anything unusual at the end of the experiment, videos received comments relating to the sincerity and realism of facial expressions, deepfakes elicited comments relating to video quality and the use of filters, and morphs elicited comments relating to the artificiality and computer-generated imagery of the expressions.

## People perceive emotions in convincing puppetry deepfakes similarly to videos

Findings suggest that deepfakes are capable of representing emotions with some accuracy. Bayesian analysis demonstrated strong evidence that videos and deepfakes were similarly intense, and moderate evidence that were similarly genuine. Our qualitative findings suggest that while participants noted some abnormalities, such as the presence of a filter, they rarely became suspicious of the deepfakes, which is consistent with research showing that high-quality deepfakes are difficult to detect (Köbis et al., 2021; Korshunov & Marcel, 2021; Tahir et al., 2021; Wöhler et al., 2021). These observations reinforce the primary findings that while deepfakes and videos display minor differences in their first and last frames, their dynamism is perceived similarly. Our findings align with recent research demonstrating that naïve participants perceive the intensity and sincerity of basic emotions depicted in face-swap deepfakes similarly to videos (Wöhler et al., 2021). We show that this holds true for whole-head, puppetry deepfakes, even though they are entirely synthesized. We also show that deepfakes can be made from established facial expression databases, using the frames from a pair of videos, and that the resulting deepfakes elicit similar behavioural responses to the originals.

## Emotions depicted in dynamic morphs are perceived differently from video recordings and deepfakes

Our participants perceived dynamic morphed emotions as less intense, while they perceived dynamic morphed happiness and sadness as less genuine compared to video recordings. Overall, our findings add to the literature showing dynamic morphs fail to elicit the same social responses as video recordings (Korolkova, 2018), and that linear (Cosker et al., 2010; Krumhuber et al., 2007; Oda & Isono, 2008; Wallraven et al., 2008), and synchronous (Curio et al., 2006) facial motion is perceived differently from naturalistic facial motion. We also show that dynamic morphs are perceived as less intense (fear, happiness, sadness) and genuine (happiness, sadness) than deepfakes, despite both being computer-manipulated, and suggest that this is due to their differences in naturalistic movement. Deepfake and videos did not differ in intensity or genuineness for any emotion tested. However, participants viewed angry videos, but not angry deepfakes, as more intense than dynamic morphed anger. This may indicate enhanced depth of anger is captured in videos. On the other hand, it is worth nothing that mean intensity ratings for angry deepfakes were higher than morphs, but this difference failed to survive Bonferroni correction. In any case, our findings demonstrate that compared to deepfakes, dynamic morphs are a less effective substitute for videos.

This study measured genuineness due to its high social value (Zloteanu et al., 2018) and to avoid leading questions about naturalism that risk revealing that some stimuli were artificially animated. However, given the expressions depicted in the ADFES database used here are posed by actors, there is no “correct” answer. Hence, emotion genuineness ratings reflect perceived differences between stimulus types rather than participants’ accuracy in deciphering social cues. Nevertheless, given that smiles are a common form of social communication (Rychlowska et al., 2017) and are the easiest expression to pose voluntarily (Jia et al., 2020), they may be more genuinely felt, or more convincingly faked. This may explain why deepfakes and videos of smiles appeared more genuine than other emotions. The absence of this effect in dynamic morphs suggests that some sincerity cues are lost in translation. Sad expressions, despite having similar genuineness ratings to anger and fear across display types, were also perceived as more genuine in videos and deepfakes compared to dynamic morphs. Smiles and sadness are connected to social bonding (Weiß et al., 2021), while anger and fear are threat-related (Pichon et al., 2009). Naturalistic facial motion may therefore be particularly important for interpreting genuine emotion from social bonding rather than threat-related expressions.

Participants often labelled dynamic morphs as “computer-generated”, which is accurate in terms of the dynamism, but not the face per se. While the intermediate frames are interpolated (and therefore do not exist in the original videos), they are more accurately described as computer-manipulated. Interestingly, deepfakes, which are wholly computer-generated, did not receive the same label. This divergence might result from the term “computer-generated,” which can suggest unrealistic, low-quality animations or avatars. This is not the case for deepfakes, where every frame is generated with intricate knowledge about the source identity’s emotional expressions, as well as intricate knowledge of how a real human (the destination) moves. It therefore appears that while deepfaked emotions are convincingly computer-generated, morphed emotions are unconvincingly computer-manipulated.

The question “did you notice anything unusual” is a semi-open-ended prompt with the option for additional information. During pilot testing, we discovered that prompts such as “describe the task” elicited fewer and less informative responses, which often centred on the strength and genuineness of emotions – data that we had already quantitatively collected. In contrast, asking about anything unusual typically elicited participants to discuss the stimulus type without being told to do so directly. Nonetheless, this question might have led participants to assume that the stimuli were computer-generated when they may not have thought so otherwise. This is supported by the fact that even video stimuli were occasionally described as computer-generated, although this may also be attributed to our editing of stimuli colour and noise to achieve texture matching (see Methods section). Despite the potential for question bias, comparing themes across the three tasks revealed a clear trend in which morphs are perceived as less realistic upon reflection.

To our knowledge, this study represents the first direct comparison of deepfakes of emotional expressions with both videos and morphs, offering valuable insights into the effects of this technology on emotional communication. While the findings are useful for research that aims to employ deepfakes in emotion perception paradigms, they also carry potentially concerning implications. The manipulative use of emotional content in advertising, politics, and social media is well documented (Flick, 2016; Kang et al., 2022; Martel et al., 2020; Otamendi & Sutil Martin, 2020), and as deepfakes become increasingly prevalent in social media and politics, their potential impact on emotion perception and social interactions should not be overlooked. Our study found that deepfakes have a similar impact on naïve participants as videos, underscoring the need for vigilance in their use by political figures and others. These implications extend beyond research to numerous domains, including entertainment, politics, and security. Future research should continue to examine the impact of deepfake technology on human perception and behaviour.

## Constraints on generality and future directions

Certain factors such as education level (Demenescu et al., 2014) and cultural background (Engelmann & Pogosyan, 2013) influence face perception. Our outreach was most successful within online student university groups, and our sample was mostly located in Australia. Thus, our sample is likely to contribute towards the overrepresentation of Western, educated, industrialised, rich, and democratic (WEIRD) populations in research (see Roberts et al., 2020). Additionally, our study can only make inferences about the perception of Northern European, Mediterranean (Turkish, Moroccan), and mixed-race faces. As the actors were culturally Dutch and emotional expressions can differ by culture (Srinivasan et al., 2016), further research on non-Western expressions is needed. Dynamic morphs are often used to examine the perception of subtle or partial expressions and should therefore be compared to video recordings of these expressions before use in face perception research. Unlike dynamic morphs, deepfakes can produce complex and conversational expressions, and expressions that contain blinks and head movements. Wöhler et al. (2021) has shown that complex expressions may be more difficult to deepfake, necessitating further comparisons as technology advances.

Posed, face-forward expressions of basic emotions were chosen as they are widely used in the literature and facilitate the standardisation of video recording lengths for comparison to dynamic morphs. However, such expressions differ from spontaneously induced expressions in their temporal features (Mavadati et al., 2016), and this may contribute to differences in how they are perceived (Krumhuber & Manstead, 2009; Zloteanu et al., 2018). Similarly, highly standardised stimuli may elicit distinct responses compared to stimuli captured under naturally occurring conditions (Dawel et al., 2022). As such, the interpretations drawn are specifically related to the simplistic expressions portrayed. Future research in this area could examine responses to spontaneous or complex emotions, those incorporating eye-movements and attention cues, and those that incorporate rigid head movements. The deepfake technology used in this paper may aid in creating such complex dynamic stimuli.

Post hoc analysis revealed that unlike videos and dynamic morphs, deepfakes were rated as slightly less intense in the second compared to the first half of the task. However, we note that the effect of the habituation was small, and much smaller than the effect size for differences between display types. In addition, the relationship between tasks did not change over time. That is; deepfakes and videos were perceived as similarly intense, and both were rated as more intense than dynamic morphs, in the first and second half of the task. Nevertheless, the habituation of deepfakes was an unexpected finding. Video recorded expressions portrayed 40 unique temporal movement patterns (one for each video), while deepfakes portrayed eight different movement patterns (one for each gender and emotion), which could have led to the observed habituation. On the other hand, dynamic morphs involved an even greater degree of repetition given they portrayed only one (linear) movement pattern but did not result in any habituation. Habituation of repeated expression dynamics is something that should be considered for future research using deepfakes, though the small effect could indicate it is not a barrier to use.

## Conclusion

In conclusion, this study aimed to investigate how people perceive emotions in video recordings, compared with two alternative approaches to creating more controlled dynamic stimuli: dynamic morphs and deepfakes. Our findings indicate that people perceive dynamic morphed emotions differently from videos and deepfakes. Specifically, they perceive dynamic morphed happiness, sadness and fear as less intense, more computer-generated, and dynamic morphed happiness and sadness as less genuine. Although it could be argued that based on the current findings face perception researchers could use morphs to measure the perception of genuineness from posed expressions of anger or fear (and avoid happiness and sadness), our overall findings suggest that dynamic morphs alter the perception of emotional expressions, hence we recommend against using them. In contrast to dynamic morphs, people appear to perceive deepfakes of basic emotions from validated databases similarly to original recordings. This similarity suggests that deepfakes may offer a more suitable standardisable stimulus type compared to dynamic morphs. Our study contributes to the growing body of research exploring the use of novel technologies and approaches in the study of human perception.

## Data Availability

All data and stimuli are available at the project’s online repository: 10.17605/osf.io/kx8h6

## Declarations

### Open practice statement

In line with Journal Article Reporting Standards (JARS; Kazak, 2018), we report how we determined our sample size, all data exclusions, all manipulations, and all measures in the study. All data and stimuli are available at the project’s online repository: 10.17605/osf.io/kx8h6. This study was not preregistered.
